# Chemical compounds from *Dictyostelium discoideum* repel a plant-parasitic nematode and can protect roots

**DOI:** 10.1371/journal.pone.0204671

**Published:** 2018-09-27

**Authors:** Yumiko F. Saito, Saki H. Miyazaki, Derek G. Bartlem, Yukiko Nagamatsu, Tamao Saito

**Affiliations:** 1 Graduate School of Science and Technology, Sophia University, Tokyo, Japan; 2 Research Faculty of Agriculture, Hokkaido University, Sapporo, Japan; 3 Institute of Environmental Science, Panefri Industrial Company, Okinawa, Japan; 4 Faculty of Science and Technology, Sophia University, Tokyo, Japan; Aarhus University, DENMARK

## Abstract

Slime mold species in the genus *Dictyostelium* are considered to have a close relationship with non-parasitic nematodes; they are sympatric in soils and can exhibit interspecific competition for food. We investigated whether this relationship extends to a plant-parasitic nematode that is active in the rhizosphere and has broad host specificity, damaging crops worldwide. Using a novel assay to examine the interaction between the cellular slime mold, *Dictyostelium discoideum*, and the plant-parasitic nematodes, *Meloidogyne* spp., we found that cellular slime molds can repel plant parasitic nematodes. Specifically, the repulsion activity was in response to chemical compounds released by cellular slime mold fruiting bodies. Under laboratory conditions, these soluble chemical extracts from fruiting bodies of *D*. *discoideum* showed repulsion activity strong enough to protect plant roots. The fruiting body cell extracts repelled but were not toxic to the plant-parasitic nematodes.

## Introduction

Cellular slime mold species in the genus *Dictyostelium* are soil microbes that feed on bacteria and yeasts. These cellular slime molds have a unique two-stage life cycle, including a uni-cellular and a multi-cellular stage. While consuming bacteria or yeasts in the soil, *Dictyostelium* spp. live as uni-cellular amoebae and increase in abundance via binary fission (vegetative-stage). Upon starvation, amoebic cells aggregate and transform into intermixed multi-cellular mounds, referred to as slugs, that differentiate into anterior prestalk and posterior prespore cells at random positions in the mound [1]. Using phototaxis and thermotaxis, slugs move toward the surface of the soil and complete cellular differentiation into the fruiting body, which form a spore head consisting of a mass of stress-resistant spores. Recently, more attention has been given to ecological studies of *Dictyostelium* species. There are several reports describing the interaction between slime mold species and bacteria [2–4]. The chemical compounds released from bacteria were identified as a predator defense mechanism [5–7].

Among other soil organisms, nematodes exist in a close relationship with cellular slime molds. This is most easily demonstrated by the fact that cellular slime mold samples isolated from soil are almost always “contaminated” with nematodes. A predator–prey relationships between *Dictyostelium* spp. and free-living nematodes was first reported in 1996 [8]. The authors found that nematodes feed on amoebae and once slime molds developed to the multicellular stage, aggregates and slugs were protected by the slime sheath. At the fruiting body stage, dauer nematode larvae climb up to the spore mass whereas adult nematodes remain in the stalk base. These data suggest that interspecific communication exists between free-living nematode and cellular slime molds in the genus *Dictyostelium*.

We focused on the relationship between cellular slime molds and plant-parasitic nematodes to understand if this microbial communication extended to non-free-living nematode species. Root-knot nematodes (RKNs) are responsible for global agricultural losses amounting to an estimated $173 billion annually [9]. Among the RKN species, *Meoidogyne incognita* is regarded as the most polyphagous and destructive. More than 700 plant species, including almost all crop species, are considered suitable hosts for *M*. *incognita* and global warming is expected to contribute to an increased infection prevalence [10]. Volatile soil fumigants that have been traditionally used as pre-planting nematocides to control root-knot nematodes are being eliminated due to their toxicity and environmental burden [9], making the control of plant-parasitic nematodes more difficult. As a result, there is an increasing interest in the agricultural industry to develop alternative nematode-controlling strategies.

We hypothesized that there might be repellent or attractant interaction between soil microbes, in this case *Dictyostelium discoideum* and *M*. *incognita*, and that might help establishing an alternative pest management strategy. Therefore, we observed interspecific chemical communication between *D*. *discoideum* and *M*. *incognita* in this study.

## Materials and methods

### Cell culture and development

The *D*. *discoideum* KAx3 strain was grown in association with *Klebsiella aerogenes* on 1/2 SM agar plates (20.9 g/L SM agar (Formedium), 11.25 g/L KH_2_PO_4_, 3.4 g/L K_2_HPO_4_, and 8.5 g/L agar) or in HL-5 liquid axenic medium (Formediun) with 150 rpm rotation at 22°C. The cells were harvested at late log stage (we call these condition of plates as “half clear”) and washed with KK2 buffer (16.5 mM KH_2_PO_4_, 3.9 mM K_2_HPO_4_ pH6.2) at least twice to eliminate bacteria or HL-5 medium completely. Washed cells of *D*. *discoideum* were plated on KK2 buffered 1.5% phytagel or gellan gum (Sigma) in 6 cm φ petri dishes (referred to as tester plates) directly or on filter paper at the density of 1 x 10^8^ cells /cm^2^. *M*. *incognita* second-stage juveniles were prepared as described previously [11].

*D*. *purpureum* was a kind gift from Dr R. Kay (MRC Laboratory of Molecular Biology, UK) and *Polysphondylium pallidum* PN500 was a kind gift from Dr P. Schaap (University of Dundee, UK). *D*. *fasciculatum* S350 was provided by NBRP-nenkin (http://nenkin.nbrp.jp). These species were grown in association with *Escherichia coli* B/r on 2–5 LP agar plate. LP agar plate was made according to the previous report with modification [12]. In brief, we used lactose instead of glucose for LP plate.

### Assessment of nematode behavior

**Fig 1:**
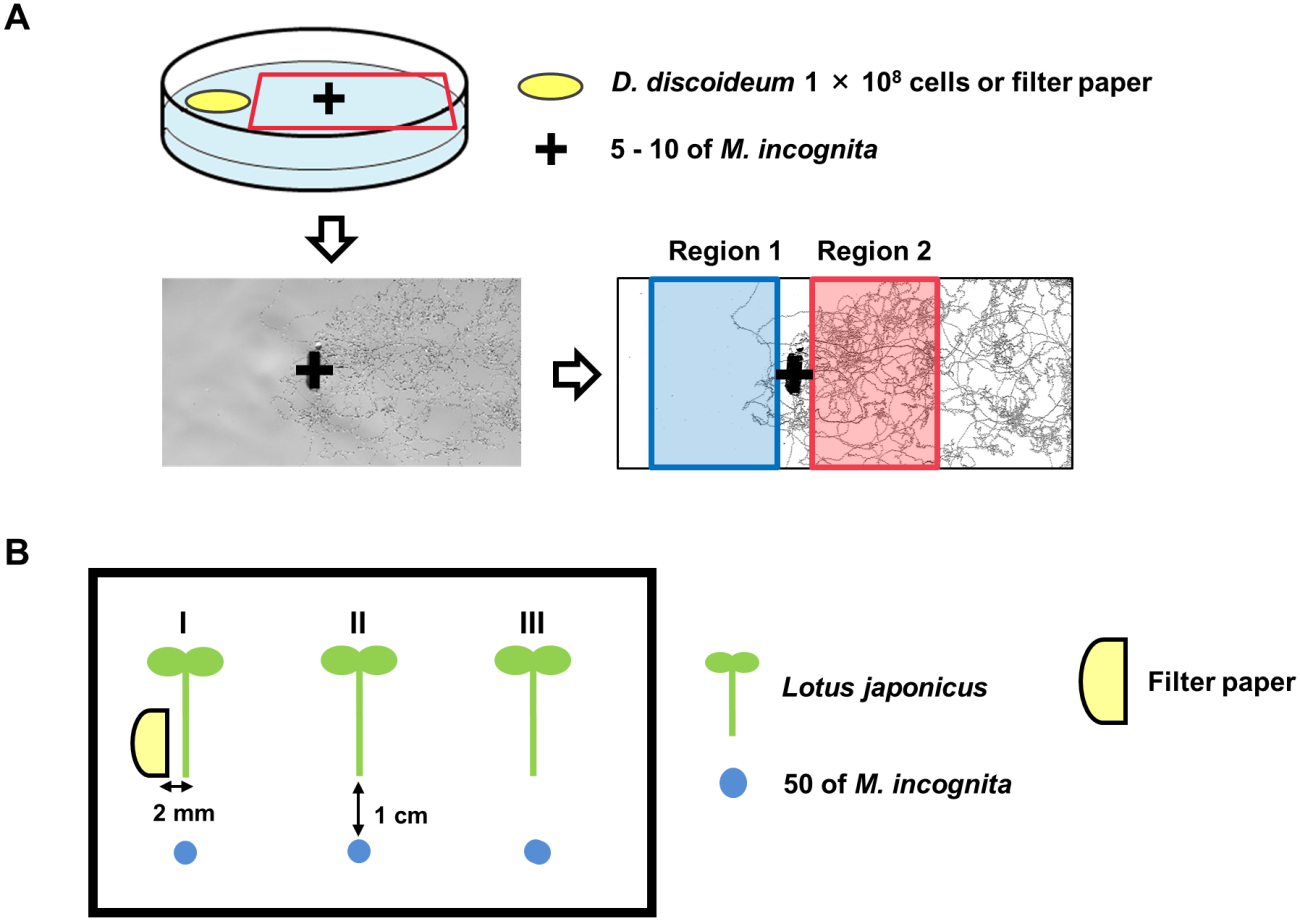
Scheme of the nematode behavior tests and plant infection test. (A) *Dictyostelium discoideum* cells or cell extract, cell-released material was placed in the yellow circle. Five to ten nematodes were placed 1.9 cm away from cells of *D*. *discoideum* and 1.2 cm away from the filter paper in the tester plate with phytagel. The deposit position of nematodes was indicated as “+”. The photograph of the tester plate was taken after 24 h and the trace of nematode movement was analyzed using ImageJ software. Trace in Region 1 indicates attraction and trace in Region 2 indicates repulsion. The trace of nematodes in each area was converted into the number of pixels, and the behavior was quantified. (B) Three *L*. *japonicas* seedlings (I-III) were placed separately in the square plastic plate with Lotus B&D medium. *Dictyostelium* cell extract was spotted and dried on semicircular filter paper. The filter paper was placed 2 mm from the side on the root tip.

Fig 1A shows the bioassay method that we designed. Cells of *D*. *discoideum* were first allowed to develop on the tester plate for 48 h to make fruiting bodies. Controls were performed by KK2 buffer. After fruiting bodies formed, 5–10 nematodes were placed in the center of the petri dish; either 1.9 cm away from *D*. *discoideum* cells located directly on the media or 1.2 cm away from filter paper-based samples when used. The nematodes were allowed to migrate for 24 h and then a snap-shot of their total movement was obtained by high-contrast imaging of the juvenile ‘trails’ in the media, captured using a microscope (Nikon AZ100 or Leica MZ 10F) fitted with a digital camera (Olympus DP26 or FLOVEL FR-400-ST).

The raw images of the nematode trails were scanned, traced using ImageJ software, and movement quantified by counting the number of pixels matching the trails within a specific region. As highlighted in Fig 1A, the relative number of trail pixels in Region 1 (attraction region) and Region 2 (repulsion region) were calculated. If more than 70% of total trace was found in either region, then this was considered as an indicator for either repulsion (Region 2) or attraction (Region 1) depending on which region was favored. Cell-released materials and cell extracts were suspended in 40% methanol. The solution or 40% methanol (control) was absorbed to the filter paper and completely air dried at room temperature for 2 h. The dried filter was used for the bioassay.

Fifty juveniles of *M*. *incognita* were placed 1 cm away from the root tip of each plant. After 2 days incubation in the plant chamber, the number of nematodes infecting the plant was counted with a microscope after acidic fuchsin staining.

### Chemical compounds released from *Dictyostelium discoideum*

Cell-released materials were prepared as described previously [13]. In brief, bacterially grown cells were harvested and allowed to develop for 3 days on filter paper placed on a stainless-steel mesh. The under surface of the mesh was in contact with phosphate buffer containing Amberlite XAD-2 resin beads to bind cell-released materials. After complete development, the beads were collected and washed briefly with water and then extracted with ethanol. The extracted materials were concentrated via rotary evaporation and eluted in 40% methanol at the concentration of 4 mg/ml.

### Cell extract from *Dictyostelium discoideum*

Cells of *D*. *discoideum* were cultured by two-member culture such that cells of *D*. *discoideum* were spread on the SM agar plate with *K*. *aerogenes*. Cells were incubated at room temperature for about one week until the cells ate the bacteria and generated fruiting bodies. When development was complete, fruiting bodies were collected and were extracted with 99.5% ethanol. The ethanol extract was filtered with filter paper (No.131 ADVANTEC), dried by rotary evaporator and eluted in 40% ethanol. The suspension was centrifuged at 15,000 g for 30 min at 4°C to acquire a supernatant. The supernatant was dried and eluted in 40% methanol at the concentration of 80 mg/ml.

### Plant infection tests

Legumes (*Lotus japonicas* MG-20) used for infection tests were kindly bestowed by Prof Kanzawa (Sophia University). Plant seeds were scarified and germinated in water with mild rotation. The germinated seeds were transferred to petri dishes with Lotus B&D medium [14] supplemented with 5 mM KNO_3_, 0.5% sucrose and solidified with 0.6% Phytagel (pH 6.8) and incubated in plant chambers. After 2 days, three *L*. *japonicas* seedlings were placed separately in the square plastic plate with Lotus B&D medium and incubated for 2 days. Fig 1B illustrates the method for plant infection tests. About fifty nematodes were spotted 1 cm away from the root tip. Plates were placed upright and incubated for 48 h in the plant chamber with overhead light. Plant II and III were set to estimate the area of cell extract affects (Intervals of each plants is 3.5 cm). The number of nematodes infecting the plant was counted with a microscope after acidic fuchsin staining [11,15].

### Statistical analysis

**Fig 2:**
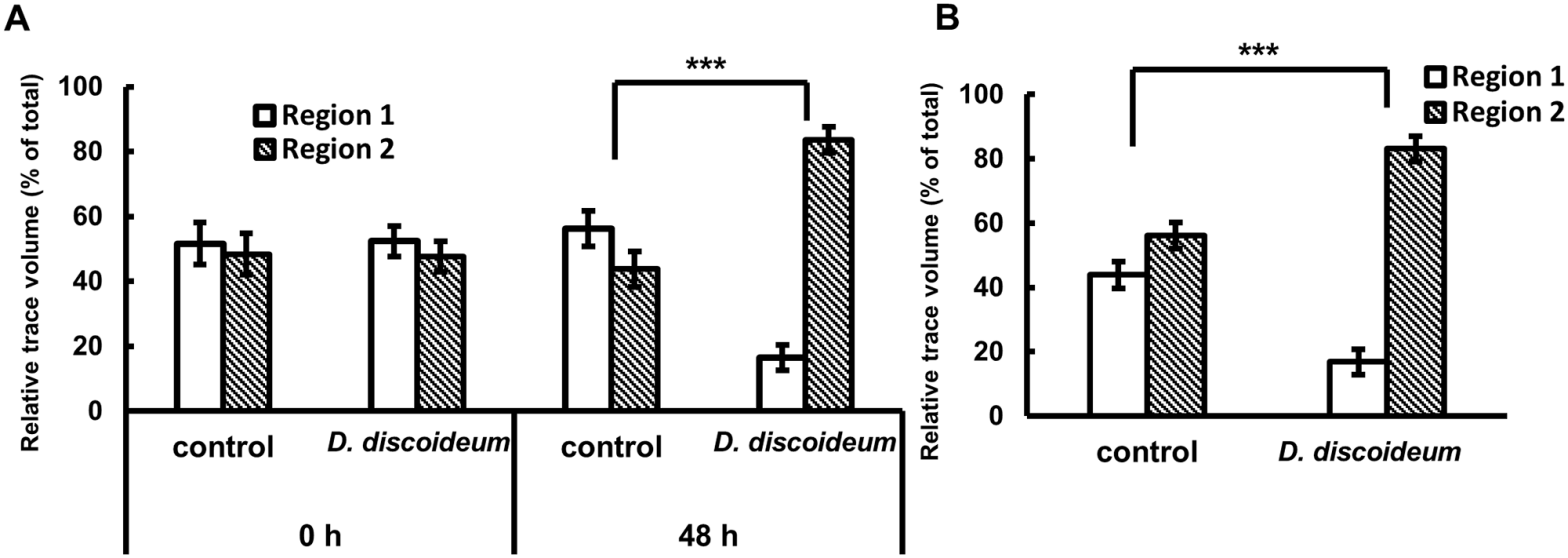
Differential responses of *Meloidogyne incognita* to *Dictyostelium discoideum*. (A) Fruiting bodies (48 h), but not vegetative cells (0 h), of *D*. *discoideum* repels *M*. *incognita*. Values are expressed as mean±s.e. of *N* ≥ 13, ***P < 0.001 versus control (Student’s t-test, unpaired, two-tailed). (B)This repulsion activity remained in the tester plate gel after the removal of fruiting bodies. Values are expressed as mean±s.e. of *N* ≥ 13, ***P < 0.001 versus control (Student’s t-test, unpaired, two-tailed). 1 x 10^8^ cells were used for each experiment.

**Fig 3:**
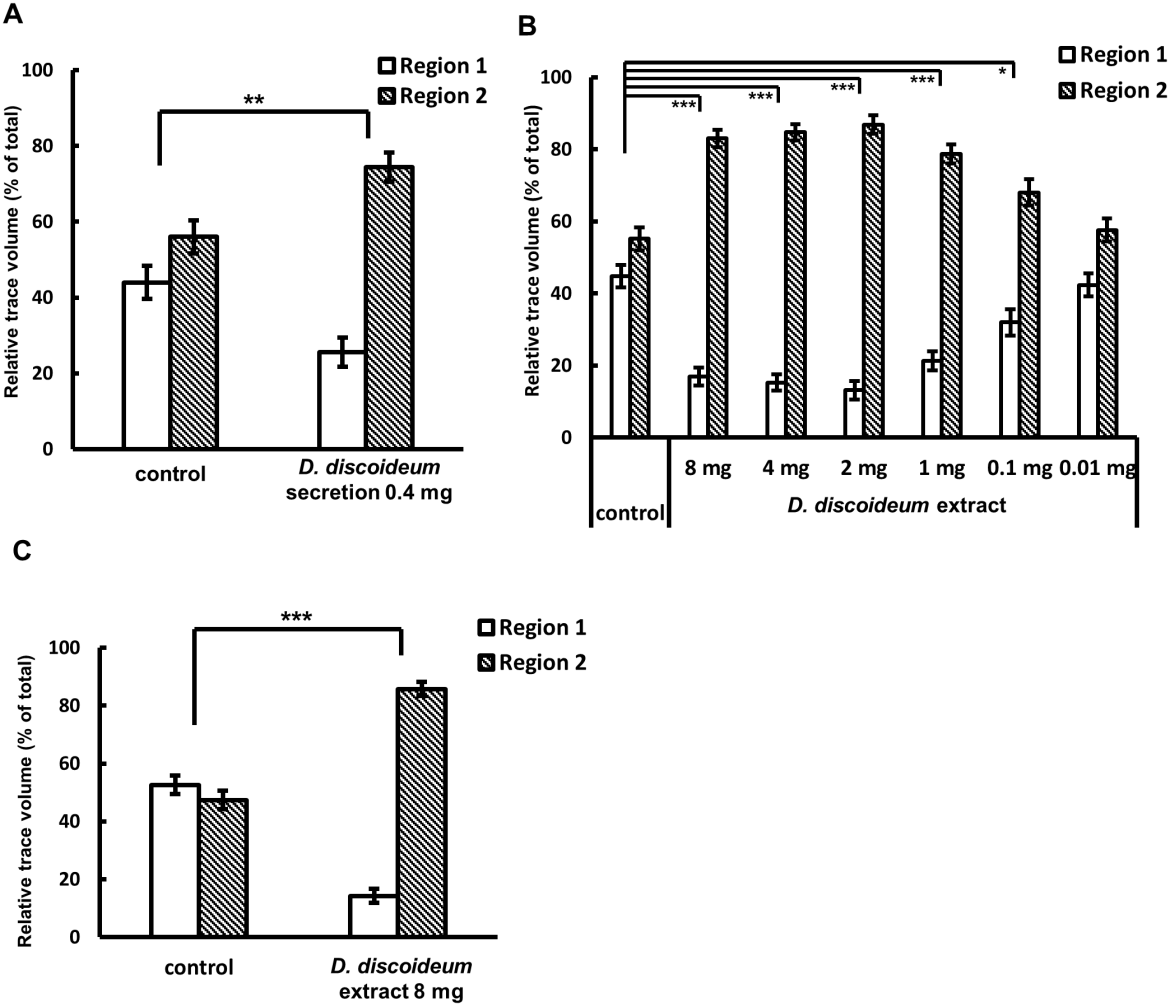
Responses of *M*. *incognita* to *D*. *discoideum* cell released materials and fruiting bodies extract. (A) 74% of total trace was found in Region 2, indicating *D*. *discoideum* cell-released materials repelled *M*. *incognita*. Values are expressed as mean±s.e. of *N* ≥ 10, **P < 0.01 versus control (Student’s t-test, unpaired, two-tailed). (B) The cell extract showed repulsion activity in a dose-dependent manner. Values are expressed as mean±s.e. of *N* ≥ 17. *P < 0.05 and ***P < 0.001 versus control (One-way ANOVA followed by Tukey-Kramer test) (C)The cell extract from axenically grown *D*. *discoideum* also repelled *M*. *incognita*. Values are expressed as mean±s.e. of *N* ≥ 16. ***P < 0.001 versus control (Wilcoxon rank sum test).

**Fig 4:**
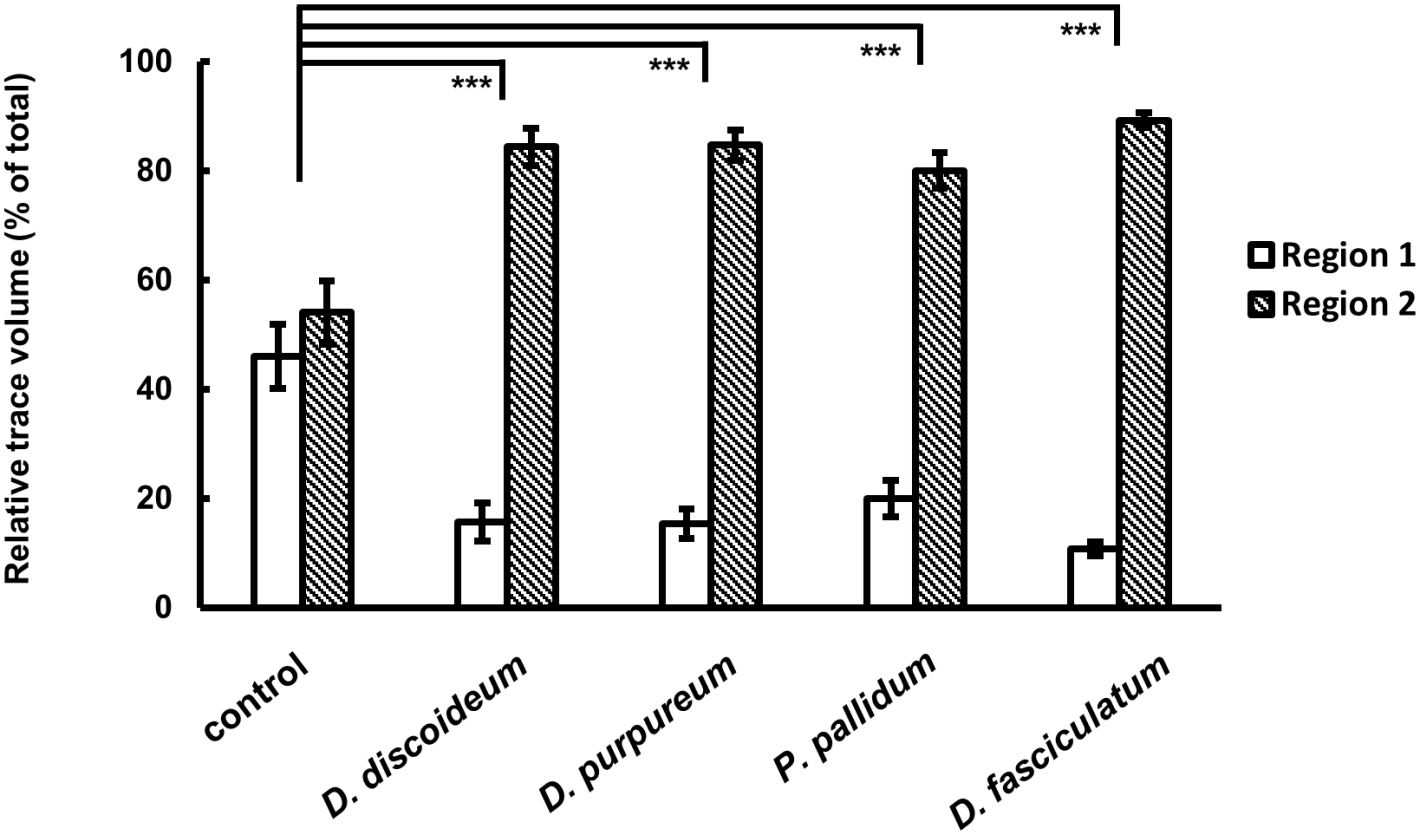
*Dictyostelum* species in different phylogenic group also repelled *M*. *incognita*. In this experiment, each Dictyostelia species was cultured with *E*. *coli* on LP agar medium. *D*. *purpureum* (group 4), *P*. *pallidum* (group 2) and *D*. *fasciculatum* (group 1) cell extracts showed the same repulsion activity with that of *D*. *discoideum* (group 4). Values are expressed as mean±s.e. of *N* ≥ 16. ***P < 0.001 versus control (Kruskal-Wallis one-way analysis of variance followed by Dunnett test).

**Fig 5:**
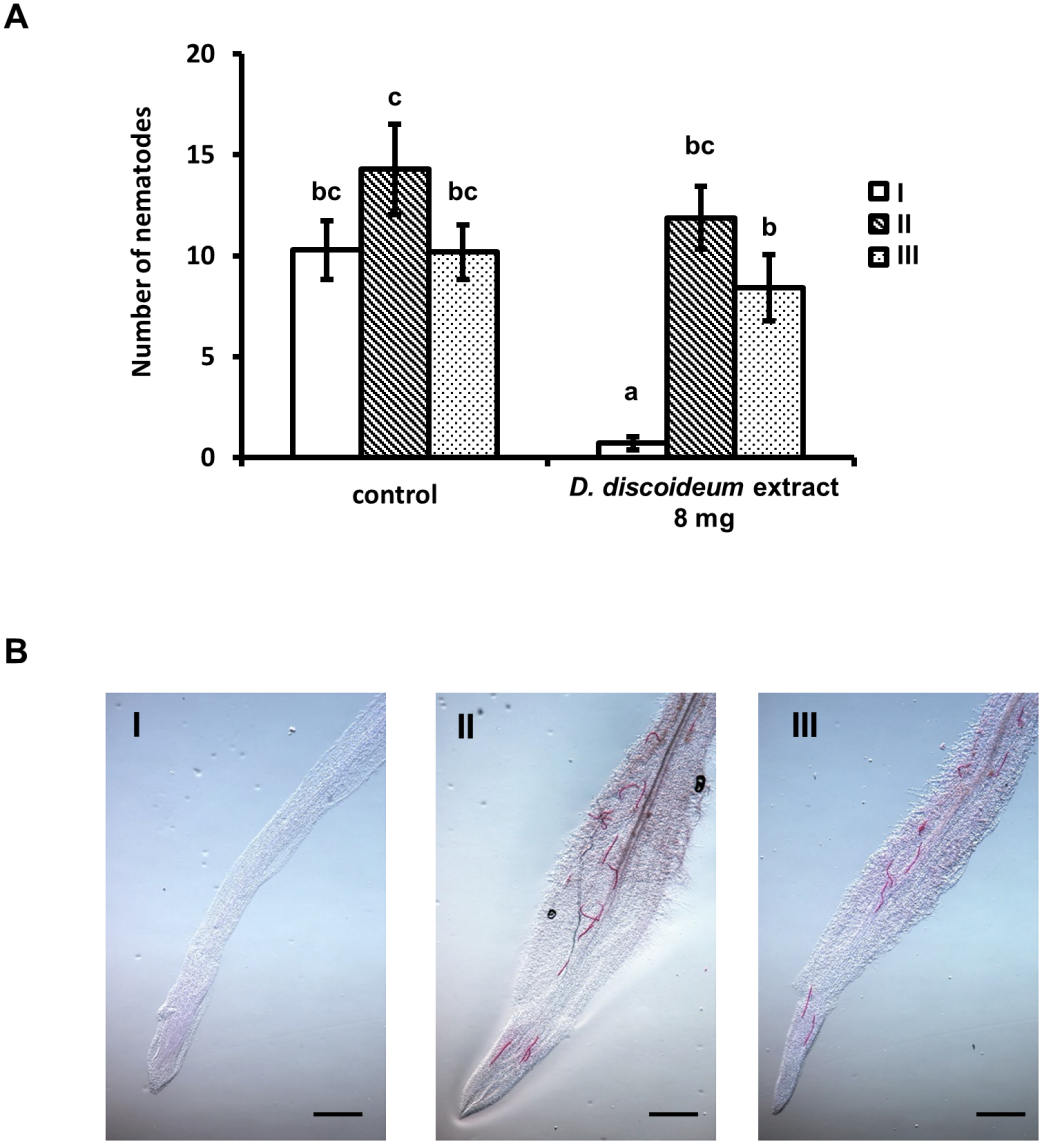
*D*. *discoideum* cell extract protects plants from *M*. *incognita* infection. (A) The number of infected RKNs of each seedling in plant infection test. Open bar indicates plant (I). Hatched bar indicates plant (II), and dotted bar indicates plant (III). Values are expressed as mean±s.e. of *N* ≥ 16. Bars with different letters denote a significant difference at *P* < 0.05 (Kruskal-Wallis one-way analysis of variance followed by Dunnett test). (B) The representative example of fuchsin staining of each plant. Bar indicates 500 μm.

Data were expressed as mean ± standard error (s.e.) and were analyzed by R version 3.5.1 [16]. The minimal number of replicate samples was 10, and each number of replicate samples was described in Figs 2, 3, 4 and 5.

The variance homogeneity and normal distribution were confirmed by F test or Bartlett test and Kolmogorov-Smirnov test. To compare values of two groups, Student’s t test was used for parametric data and Mann-Whitney U test was used for nonparametric data. One-way ANOVA followed by Tukey-Kramer test (for parametric data) and Kruskal-Wallis one-way analysis of variance followed by Dunnett test (for nonparametric data) were performed for multiple comparisons. For all statistical analyses, level of significance was set at *P* < 0.05.

## Results and discussion

Kessin *et al*. (1996) suggested that there was a predator–prey relationship between cellular slime mold species in the genus *Dictyostelium* and the free-living nematode, *Caenorhabditis elegans* [8]. Due to the large number of potential secondary metabolites produced by *D*. *discoideum* [17,18] and that the soil can be considered a battle field of chemical warfare between microorganisms, we were interested in the chemical ecology of cellular slime molds and nematodes. We focused on the relationship between *D*. *discoideum* and the plant-parasitic root-knot nematode, *M*. *incognita*, that damages crops worldwide.

### Fruiting bodies repel plant-parasitic nematodes

We first examined the behavior of *M*. *incognita* in the presence of *D*. *discoideum* in the same petri dish. Fig 2A shows the result of behavioral analysis of nematodes in the presence of *D*. *discoideum* cells. The nematode movements in the presence of vegetative-stage cells (indicated as 0 h) of *D*. *discoideum* were almost the same as that of controls. This indicates that nematodes moved freely in the petri dish in the presence of vegetative cells and there was no evidence for attraction or repulsion. For the case of fruiting bodies (indicated as 48 h), *M*. *incognita* preferentially moved toward Region 2 in the tester plate, which indicated a repulsion from *D*. *discoideum* fruiting bodies. To examine if this repulsion resulted from chemical compounds released from *D*. *discoideum*, vegetative cells were inoculated on filter paper placed directly on the tester plate media, which would allow released compounds to transfer to the media and incubated for 48 h until they generated fruiting bodies. The filter paper with fruiting bodies was then removed from the tester plate, removing all cellular material from the surface of the media. *M*. *incognita* juveniles placed on the tester plate after removal of the filter paper retained their preferential movement toward Region 2 (Fig 2B), consistent with the hypothesis that *D*. *discoideum*-released chemicals remaining in the gel were responsible for the nematode repelling activity.

### Cell-released materials and cell extracts repel *M*. *incognita*

The above data indicated the presence of chemical communication between *D*. *discoideum* and *M*. *incognita*. To examine this further, we first used cell-released materials from *D*. *discoideum* for the bioassay. As shown in Fig 3A, 74% of total trace was found in Region 2, indicating *D*. *discoideum* cell-released materials repelled *M*. *incognita*. In this experiment, we used 0.4 mg of cell-released materials is equivalent to 7–10 x 10^8^ cells, which is much more than what we used in the experiment shown in Fig 2. This might mean that the extraction method with XAD-2 resin which preferentially absorb hydrophobic organic compounds was not suitable to collect the compounds with repulsion activity. This may reflect the chemical nature of repellent(s) produced by *Dictyostelium* fruiting bodies. Therefore, we next examined if we can extract the compounds with repulsion activity from *D*. *discoideum* fruiting bodies directly.

For this purpose, we prepared the cell extract and examined repulsion activity. Fig 3B shows that the *D*. *discoideum* cell extract repelled *M*. *incognita* in a dose-dependent manner. One mg of cell extract is equivalent to 1 x 10^8^ cells and the repulsion activities shown in Fig 2A (48 h *D*. *discoideum*) and in Fig 3B (1 mg) are almost the same. This indicates that the method we used to prepare cell extract is adequate for detecting the repulsion activity from the cell. In the presence of the highest concentration of 8 mg cell extract (Fig 3B), nematodes moved to Region 2 and were still alive after 24 h (data not shown). This indicated that the cell extract repels but is not toxic to *M*. *incognita* at this concentration.

As the above experiments utilized bacterially grown *D*. *discoideum* cells for the preparation of the cell extract and cell-released materials, we could not eliminate the possibility that an unknown chemical compound(s) contaminant from the feeding bacteria caused the repulsion activity. To examine this, we used axenically grown *D*. *discoideum* cells for the preparation of cell extract and found that the cell extract from axenically grown *D*. *discoideum* fruiting bodies retained the *M*. *incognita* repelling activity (Fig 3C). These data strongly indicate that *D*. *discoideum* releases chemical compound(s) that repel *M*. *incognita*. We also confirmed similar results with a related RKN species, *Meloidogyne hapla* (S1 Fig), suggesting that this activity is not limited to *M*. *incognita* and likely effective against a broader range of plant parasitic nematodes.

The molecular phylogeny of Dictyostelia based on ribosomal RNA and α-tubulin data sets indicates Dictyostelia is divided into 4 major groups, and groups 1 and 2 contain the most ancient taxa [19]. *D*. *discoideum* belongs to group 4 which is the most evolved group. To address if this repulsion activity against *M*. *incognita* is common in Dictyostelia species, we selected two other species that belong to groups 1 and 2, and one additional group 4 species was also tested. Fig 4 indicates the result of four different Dictyostelia species. All cell extracts of the selected species showed similar repulsion activity, indicating that the chemicals that repel *M*. *incognita* are likely shared among Dictyostelia species.

We do not understand how this phenomenon was selected for. One possible explanation for the ecological mechanism for the *M*. *incognita* repulsion activity by *Dictyostelium* is that might be based on different vertical distributions of each microbe in the soil. *M*. *incognita* is a subterranean microbe that infects plants through the extended region behind the root tips. RKNs are widely distributed vertically, and some are distributed to a depth of 35 cm [20,21]. On the other hand, cellular slime molds are distributed on the surface layer of the soil, and it has been reported that fruiting bodies do not form below 1.5 cm from the soil surface [22]. The fruiting body formation is a survival strategy for cellular slime molds [23–25]. In order to disperse spores to new feeding site, cellular slime molds take advantage of insects, birds, and nematodes [24,26,27]. Free living nematodes that share food bacteria with *Dictyostelium* has been reported to be attracted by *Dictyostelium* fruiting bodies and to spread spores [8]. In fact, *C*. *elegance* was attracted by *Dictyostelium* cell extract (data not shown). From this point of view, RKNs may not be favorable spore vectors and the immobile fruiting bodies appear to use chemical compounds to ensure these nematodes are excluded during this developmental stage.

### Cell extracts protect plant roots from *M*. *incognita* infection

We found that cell extracts of *D*. *discoideum* contain a repellent to RKNs. As *M*. *incognita* is one of the more destructive agricultural soil pests, we assessed if cell extracts of *D*. *discoideum* were able to protect plants from infection. Plant infection tests confirmed a significant plant protection activity by the *D*. *discoideum* cell extracts (Fig 5A), with almost no detectable presence of infective *M*. *incognita* juveniles in roots located in proximity to the cell extract, that is plant I (Fig 5B). On the other hand, plant II and III did not show significant difference versus control. It indicates that cell extract affection area is limited only around plant I in this case.

Our results have important implications for novel nematode control strategies. Chemical communication between RKNs and plants has recently been reported. Lauric acid in crown daisy root exudates attracts and repels *M*. *incognita* in a concentration-dependent manner [28]. Thymol from roots of *Capsicum annum* repels *M*. *incognita* [29]. These results could help create novel strategies for pest management. A detailed analysis of the *D*. *discoideum* chemical compound(s) that repels *M*. *incognita* would be the next step of this study.

## Conclusion

Our findings indicate that parasitic nematodes and cellular slime molds exhibit an interspecific chemical communication. The chemical compounds released from the fruiting bodies of *D*. *discoideum* were active against plant-parasitic nematodes and protected plants from infection. Our results reveal an exciting and novel avenue for management of nematode crop pests.

## Supporting information

S1 Fig Responses of *M*. *hapla* to *D*. *discoideum* cell released materials. *D*. *discoideum* cell-released materials repelled *M*. *hapla*. Values are expressed as mean±s.e. of *N* ≥ 32, ***P < 0.001 versus control (Student’s t-test, unpaired, two-tailed).
(TIF)
