# Supplementary figures and images for: Chemical compounds from Dictyostelium discoideum repel a plant-parasitic nematode and can protect roots
Source: PLoS One. 2018 Sep 27;13(9):e0204671. doi: 10.1371/journal.pone.0204671 (PMC6160129; doi:10.1371/journal.pone.0204671)

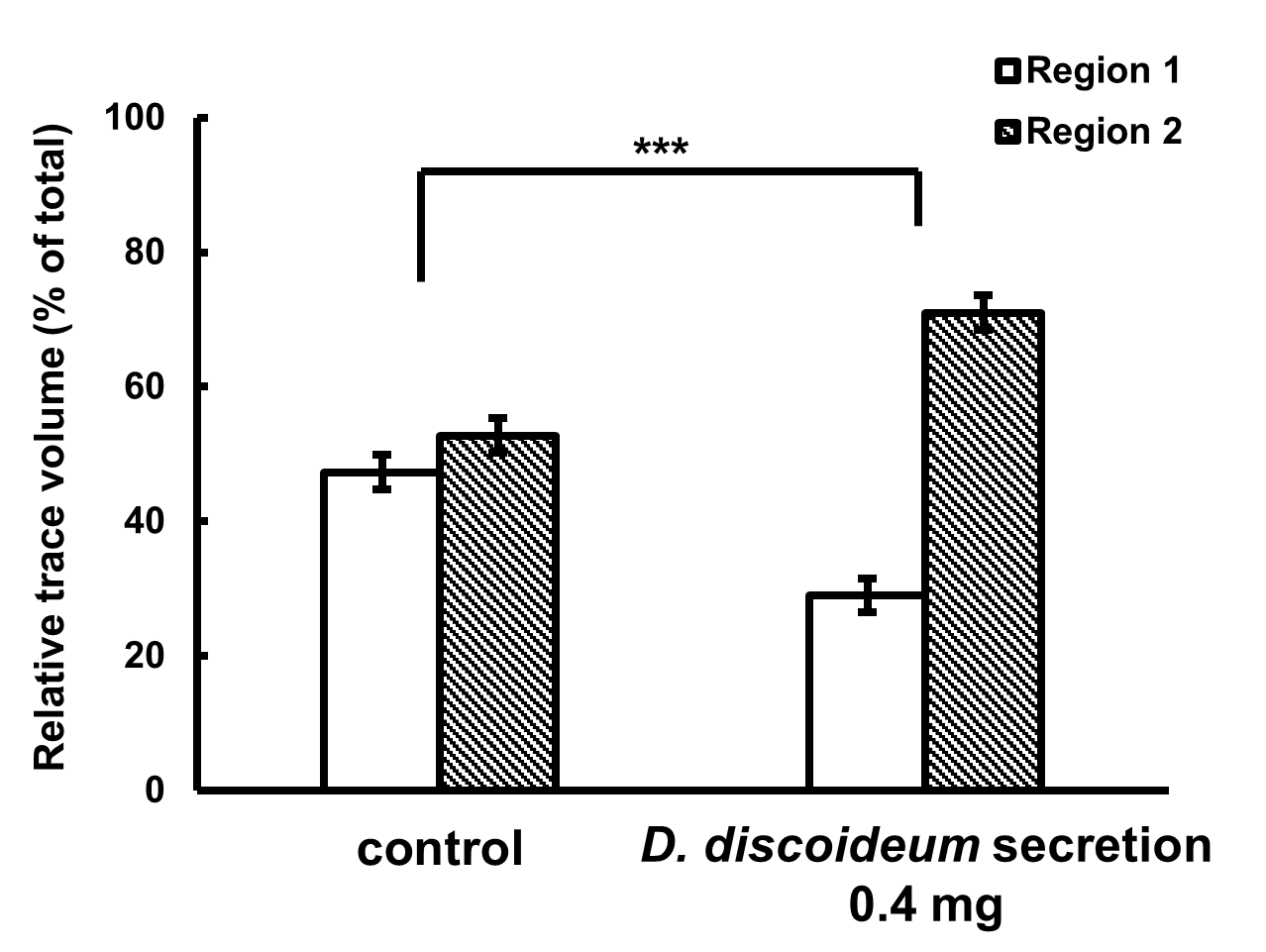

Supplement: S1 Fig — D. discoideum cell-released materials repelled M. hapla. Values are expressed as mean±s.e. of N ≥ 32, ***P < 0.001 versus control (Student’s t-test, unpaired, two-tailed). (TIF) [file pone.0204671.s001.tif]
